# Progesterone levels on the day of the β-hCG test predict pregnancy outcomes in FET cycles

**DOI:** 10.1038/s41598-025-30902-9

**Published:** 2026-01-19

**Authors:** Seçil İrem Arık Alpçetin, Tamella Taghiyeva, Erhan Demirdağ, Duru Erdem, Münire Funda Cevher Akdulum, Pınar Çalış Tokdemir, Nuray Bozkurt, Ahmet Erdem, Mehmet Erdem

**Affiliations:** 1 Department of Obstetrics and Gynecology, Gazi University, Ankara, Turkey; 2 NovaArt Reproductive Healths and Fertility Center, Ankara, Turkey

**Keywords:** Frozen-thawed embryo transfer, Progesterone, Pregnancy outcome, Luteal phase support, β-hCG test day

## Abstract

Serum progesterone (P) levels are critical for endometrial receptivity and implantation in frozen-thawed embryo transfer (FET) cycles. However, the prognostic role of P levels measured on the day of the β-human chorionic gonadotropin (β-hCG) pregnancy test has not been fully elucidated. This study aimed to evaluate the association between β-hCG day serum P levels and pregnancy outcomes in FET cycles. This retrospective cohort study included 621 FET cycles performed between January 2023 and December 2024, of which 79.5% were conducted using hormone replacement therapy (HRT) protocols and 20.5% using natural cycle (NC) protocols. Serum P levels were measured on the day of the β-hCG pregnancy test. Receiver operating characteristic (ROC) curve analysis was used to determine protocol-specific P thresholds for predicting ongoing pregnancy (OPR). Ongoing pregnancy was defined as a viable intrauterine pregnancy confirmed by ultrasound at or beyond 12 weeks of gestation. Multivariable logistic regression was applied to identify independent predictors of OPR. ROC analysis identified optimal P thresholds of 15.5 ng/mL in NC cycles (AUC 0.821) and 14.15 ng/mL in HRT cycles (AUC 0.595). Overall, 44% of patients had serum P levels below the protocol-specific threshold. OPR was significantly higher in patients with P levels above the threshold (NC: 63.0% vs. 12.8%; HRT: 48.1% vs. 31.9%; *p* < 0.001). Multivariable regression demonstrated that younger maternal age and higher β-hCG day P levels independently predicted OPR. In HRT cycles, blastocyst-stage transfer was also significantly associated with improved outcomes (OR = 0.27, 95% CI 0.13–0.59; *p* < 0.05). Serum P levels measured on the day of the β-hCG test are significantly associated with pregnancy outcomes in both HRT and NC FET cycles. Routine monitoring of late luteal P levels and individualized luteal phase support strategies may enhance clinical success rates.

## Introduction

The frozen-thawed embryo transfer cycles in assisted reproductive technology (ART) programs have increased significantly due to the advancements in cryopreservation and vitrification technologies^1^. The “freeze-all” strategy is increasingly preferred by clinicians for several reasons, including the avoidance of ovarian hyperstimulation syndrome (OHSS) risk, enabling preimplantation genetic testing (PGT), reducing the need for repeated controlled ovarian hyperstimulation –in vitro fertilization (IVF) cycles, and creating an oocyte and embryo pool for patients with diminished ovarian reserve^2^.

Progesterone plays a vital role in maintaining pregnancy and promoting immunologic tolerance^3^; its deficiency may lead to implantation failure, recurrent pregnancy loss, or preterm delivery. During the window of implantation, P4 triggers decidualization, leading to essential histological and transcriptomic changes. In spontaneous conception, P4 levels increase from around 1–2 ng/mL at the luteinizing hormone (LH) surge, reaching a stable range of 10–35 ng/mL within a week and remaining at this level until placental P4 production becomes predominant^4^. P4 deficiency can negatively impact implantation success, making adequate luteal support essential in assisted reproduction. As part of endometrial preparation strategies in frozen embryo transfer (FET) cycles, luteal phase support (LPS)—including natural cycles (NC), modified natural cycles with human chorionic gonadotropin (hCG) triggering, and hormone replacement therapy (HRT) cycles—aims to optimize implantation conditions^5^. In HRT-FET cycles, intensive LPS is necessary due to the absence of a corpus luteum, which may impact the secretion of angiogenic factors and perinatal outcomes^6,7^.

Low mid-luteal serum progesterone levels prior to or during transfer may negatively impact FET outcomes^8–10^. A 2021 meta-analysis evaluated serum progesterone levels measured before, during, and after ET. Many of these studies defined a progesterone threshold of < 10 ng/mL, reporting significantly higher clinical pregnancy and live birth rates in patients with levels above this cut off^11^. In our study, we adopted this 10 ng/mL cut off on the day of ET and implemented a rescue protocol with additional progesterone supplementation for patients below this level. In a previous study, we found that 15.8% of patients receiving micronized vaginal progesterone (MVP) alone and 8.9% of those receiving MVP plus SCP had serum P4 levels < 10 ng/mL and individualizing luteal phase support based on serum progesterone levels on the day of ET in HRT-FET cycles may improve pregnancy outcomes, either by doubling the vaginal dose or by increasing the SC dose in combined MVP plus SC protocols^12^.

Individualizing P4 doses during the mid-luteal phase before embryo transfer (ET), targeting a serum threshold of > 9–10 ng/mL, is crucial for optimal LPS and for rescuing cycles with insufficient P4 supplementation^13–15^.

In HRT-FET cycles that result in pregnancy, P4 supplementation is continued until placental autonomy is established^16^. While P4’s role during the luteal phase is well documented, its crucial function in implantation suggests that serum P4 levels in early pregnancy might impact pregnancy outcomes. Studies have shown that mean P4 levels in first trimester are significantly higher in pregnancies that progress to term, whereas lower serum P4 levels are associated with threatened miscarriage and an increased risk of pregnancy loss^17^. However, only a limited number of studies have investigated the correlation between luteal P4 levels after ET and serum P4 levels on the day of the pregnancy test in pregnant and nonpregnant patients undergoing HRT-FET cycles.

Different serum P4 thresholds on the day of the pregnancy test in HRT cycles have been identified in two studies^18,19^. One-third of patients were found to have P4 levels below the cutoff of 10.6 ng/ml on the day of the hCG test, despite receiving additional P4 supplementation due to low levels on the day of ET. Moreover, the likelihood of detecting sub-threshold P4 levels on the day of the hCG test decreases as serum P4 levels on the day before ET increase^18^. In another study, a serum P4 level above the threshold of 11.0 ng/ml(35 nmol/L) on the day of the pregnancy test was positively associated with ongoing pregnancy rates^19^. To our knowledge, serum P4 levels on the day of the pregnancy test have not been previously investigated in natural FET cycles.

This study aimed to investigate the role of P4 beyond the traditional luteal phase window, particularly on the day of the pregnancy test, in patients undergoing either HRT or natural FET cycles with a rescue protocol based on P4 levels on the day of ET, in which the P4 dose was adjusted accordingly. Additionally, we sought to examine the relationship between P4 levels on the day of ET and those measured on the pregnancy test day.

## Material and methods

This retrospective study was conducted at the Novaart IVF and Women’s Health Center between January 2023 and December 2024. The study protocol was reviewed and approved by the Gazi University Ethics Committee on February 13, 2024 (Approval No: 2024–336), and the ethical approval document has been provided as a supplementary file.

This study included infertile patients undergoing FET cycle. Clinical data were collected from the initiation of FET preparation through treatment completion and follow-up. Information was obtained from institutional databases and included comprehensive patient histories, clinical examination results, infertility evaluations, and details of the cryopreserved embryo cycles.

FET treatment protocols, hormonal monitoring during the FET cycle (including serum estradiol (E2), LH, P4, and β-hCG), endometrial thickness on the day of P4 initiation, the number and developmental stage of transferred embryos, and pregnancy outcomes were systematically recorded.

### Patient selection criteria and treatment protocols

The study included infertile patients aged 20–48 years who were referred to our clinic and scheduled for FET. Patients who completed FET cycles and met the following inclusion criteria were enrolled: body mass index (BMI) < 40 kg/m^2^ and absence of systemic diseases. All patients used their own embryos. One or two good-quality embryos (cleavage- or blastocyst-stage) were transferred in patients demonstrating a normal endometrial pattern (triple-line appearance) and an endometrial thickness > 6.5 mm.

Patients with untreated major uterine anomalies or intrauterine lesions (e.g., polyps, fibroids, or hydrosalpinx) were excluded. Cycles were also excluded if canceled due to premature progesterone elevation before luteal support, uterine bleeding, endometrial abnormalities (such as thin or echogenic endometrium), uterine peristalsis detected by real-time ultrasonography, intrauterine fluid accumulation, low serum estradiol levels after exogenous estrogen administration, or other non-clinical reasons (e.g., COVID-19 infection or patient request).

Endometrial preparation protocols were individualized according to ovulatory status. NC protocols were preferred for regularly ovulating patients, while HRT protocols were applied in anovulatory or irregular-cycle patients. Both cleavage-stage and blastocyst-stage embryos were included in the study.

### Natural cycle frozen-thawed embryo transfer (NC-FET) protocol

On day 3 of the menstrual cycle, transvaginal ultrasonography (TVUS) was performed, and patients without follicular cysts were included in the treatment. If residual follicular structures were detected, serum E2 and P4 levels were measured. Patients with hormonal activity (E2 > 75 pg/mL and P4 > 1 ng/mL) were excluded from the natural cycle protocol. Eligible patients were monitored using transvaginal ultrasound and serial hormone measurements.

Baseline monitoring was conducted on cycle days 8 to 10, individualized according to each patient’s cycle length. Dominant follicular growth was assessed by ultrasound every 2–3 days. In patients with dominant follicles measuring ≥ 12–14 mm, serum E2, LH, and P4 levels were assessed to monitor follicular development and estimate the timing of ovulation. Once the dominant follicle reached a diameter of 14–16 mm, daily ultrasound and hormonal monitoring were initiated (Orvieto, Morag et al^20^.). Ovulation was confirmed by ultrasonographic evidence of follicular rupture, along with a subsequent rise in serum progesterone levels (> 1 ng/mL) the following day. Cleavage-stage embryos were thawed and transferred on day 4 post-ovulation (corresponding to P + 4), and blastocyst-stage embryos on day 6 post-ovulation (P + 6).

Serum P4 levels were measured on the day of ET. Patients with P levels below 10 ng/mL received additional progesterone supplementation, while those with levels below 3 ng/mL had their cycles canceled.

### Hormone replacement therapy frozen-thawed embryo transfer protocol

Patients without follicular cysts were included in the treatment after TVUS assessment. A treatment initiation decision was made for patients who met the inclusion criteria, similar to those in the NC.

Eligible patients started oral 2 mg estradiol valerate (Estrofem, Novo Nordisk, Turkey) 2–4 times daily from cycle day 3. Patients were re-evaluated 8–10 days later with TVUS to assess endometrial thickness and serum hormone levels. If endometrial thickness was ≥ 6.5 mm, serum E2 > 150 pg/mL, and P4 < 1 ng/mL, P4 supplementation was initiated. The regimen included 25 mg subcutaneous progesterone (SCP) injection once daily (Progestan Dex 25 mg, Koçak Farma, Turkey) and 400 mg micronized P4 capsules (Progestan, Koçak Farma, Turkey) administered vaginally twice daily.

For patients with serum E2 levels < 100 pg/mL, supplemental transdermal estradiol patches (Climara 3.9 mg/12.5 cm^2^, Bayer, Turkey) were applied every other day. E2 levels were reassessed 4–6 days later, and if no increase was observed, the cycle was canceled. If P4 levels exceeded 1 ng/mL, indicating spontaneous ovulation, the cycle was also canceled.

ET was performed 4 to 6 full days after the initiation of progesterone, depending on the embryo’s developmental stage. Serum P4 levels were measured on the day of ET. Patients with P4 levels below 10 ng/mL received supplemental SCP with the dose doubled if needed.

### Embryo thawing and transfer procedure

The vitrification method was used for cryopreservation in all FET cycles. All embryos were fertilized using Intracytoplasmic Sperm Injection (ICSI) within the same clinic and were derived from the same patient’s fresh IVF cycles. Embryos were thawed on the day of the planned transfer and evaluated for morphology and cell number.

Embryo morphology was assessed using the Alpha/ESHRE criteria for day 3 embryos and the Gardner–Schoolcraft grading system for day 5 blastocysts, as outlined in The Istanbul Consensus Workshop on Embryo Assessment^21,22^. ET was performed under ultrasound guidance using a soft transfer catheter (Allwin Pro ECHO Transfer Catheter, USA).

One or two embryos were transferred, and hormonal therapy was continued after ET. Patients undergoing either natural or HRT cycle FET with normal P levels did not receive additional hormonal supplementation. In both treatment protocols, pregnancy status was evaluated by measuring serum β-hCG and P4 levels—12 days after transfer for cleavage-stage embryos (day 3 transfer) and 10 days after transfer for blastocyst-stage embryos (day 5 transfer). Patients whose serum P4 measurements fell outside the scheduled assessment window were excluded from the study. For patients receiving exogenous estrogen and progesterone, hormonal therapy was continued until the 10th week of gestation.

### Main outcomes

The primary outcome of this study was to determine the threshold serum progesterone (P4) level on the day of the β-hCG test that predicts ongoing pregnancy. Clinical pregnancy was defined as the presence of a gestational sac with fetal heartbeat on transvaginal ultrasound at 6–7 weeks’ gestation. Ongoing pregnancy was defined as a viable intrauterine pregnancy confirmed by ultrasound at or beyond 12 weeks’ gestation. Live birth was defined as delivery of a viable infant after ≥ 24 weeks of gestation. Biochemical pregnancy loss was defined as an early pregnancy failure occurring before ultrasound confirmation of a gestational sac. Miscarriage referred to the loss of a non-viable intrauterine pregnancy before 20 weeks of gestation, while ongoing pregnancy was defined as a viable pregnancy extending beyond 12 gestational weeks. Secondary outcome meassure was to identify independent predictors of ongoing pregnancy through multivariable logistic regression analysis, including variables such as progesterone levels, patient age, endometrial preparation protocol, and embryo stage at transfer*.*

### Statistical analysis

Statistical analyses were performed using SPSS (version 21.0; IBM Corp., Chicago, USA)**.** Descriptive statistics, frequency tables, and mean ± standard deviation (SD) were used to summarize the data. A *p*-value < 0.05 was considered statistically significant.

For normally distributed variables, parametric tests were applied. Group comparisons were conducted using the independent samples t-test. For non-normally distributed variables, non-parametric methods were used, including the Mann–Whitney U test for comparing two independent groups**.** Pearson correlation analysis was conducted to assess the relationships between selected variables. Categorical variables were analyzed using the Chi-square test**.** Since multiple cycles from the same patients were included, a generalized estimating equation (GEE) regression model was used to identify factors affecting the threshold values. To assess the predictive value of P4 levels on the day of β-hCG testing, receiver operating characteristic (ROC) curve analysis was performed for all FET protocols, both combined (NC + HRT-FET) and separately.

## Results

This study included 621 FET cycles from 504 patients. Of these, 79.5% were performed using a HRT protocol, while 20.5% followed a natural cycle protocol. Rescue progesterone supplementation was administered in 11% of NC. In HRT cycles, 3% of patients with serum progesterone levels below 10 ng/mL on the day of embryo transfer received additional supplementation. Biochemical, clinical and ongoing pregnancy rates, live birth rates were 54.7%, 46.1%, 42.4% and 36.7% of all cycles, respectively.

Mean serum P4 levels on pregnancy test day were 19.5 ± 11.9 ng/mL overall, with natural cycles demonstrating higher concentrations (22.3 ± 12.3 ng/mL) compared to HRT cycles (18.7 ± 11.7 ng/mL). In HRT and natural FET cycles, ongoing pregnancy rates increased with higher P4 levels measured on the day of β-hCG testing when analyzed by percentiles and quartiles (*p* < 0.001 and *p* < 0.05, respectively; Table 1). There was a statistically significant weak positive correlation between serum P4 levels and β-hCG values on the day of testing (r = 0.184, *p* < 0.001).

**Table 1:** Ongoing pregnancy rates according to serum progesterone levels measured on β-hCG day in FET-HRT cycles, categorized by percentiles.

		< 25% IQR	25–50% IQR	50–75% IQR	> 75% IQR	*p value*
NC	P4 on ET day (ng/mL)	< 12	12–19.6	19.6–29.5	> 29.5	< 0.001
	OPR (%)	10.0	33.3	75.8	77.4	
HRT	P4 on ET day (ng/mL)	< 11.8	11.8–14.7	14.7–21	> 21	< 0.05
	OPR (%)	28.6	42.3	42.1	49.6	

P₄ levels are grouped by interquartile ranges (*IQR*), and ongoing pregnancy rates (*OPR*) are presented as percentages within each quartile. Statistically significant differences across groups were observed in both protocols (*p* < 0.05).*HRT*: Hormone Replacement Therapy *NC*: Natural Cycle *P4*: Progesterone *ET*: Embryo Transfer.

### Estimation of cut off P4 levels to predict ongoing pregnancy in natural FET cycles

**Fig. 1:**
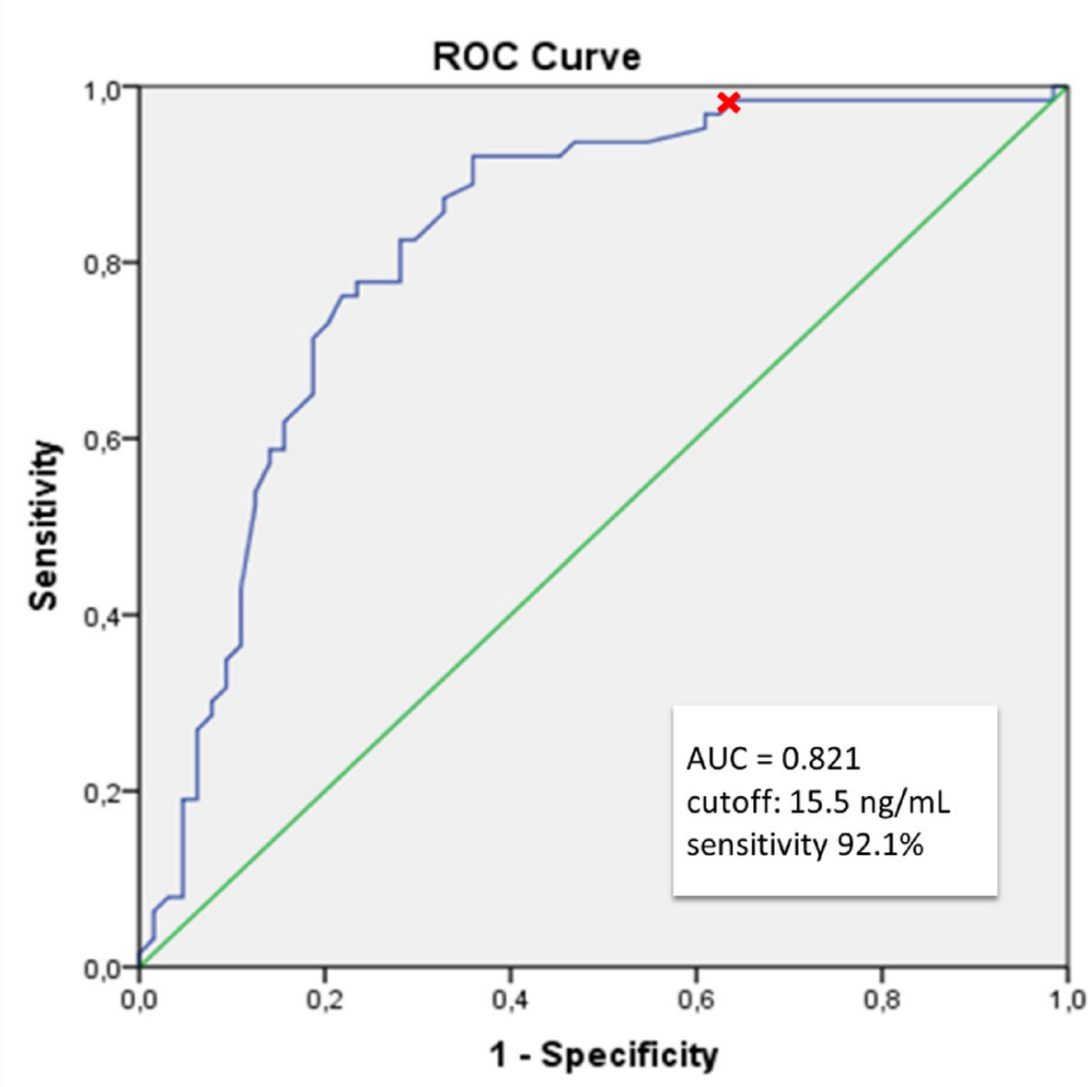
ROC analysis of serum progesterone levels on β-hCG day for predicting ongoing pregnancy in NC-FET cycles.

In patients undergoing a natural FET cycle, ROC curve analysis was performed to determine the optimal threshold value of serum P4 levels on the day of the pregnancy test in predicting ongoing pregnancy. The area under the curve (AUC) was 0.821 (CI 0.745–0.897, *p* < 0.001). A P4 threshold of 15.5 ng/mL was identified, yielding a sensitivity of 92.1% and a specificity of 64.1% (Fig. 1). In the natural cycle group, 47% of patients had serum P4 levels below the threshold.

Patients were stratified based on the P4 threshold value of 15.5 ng/mL, and comparisons of patients’ demographic and cycle characteristics based on this threshold are presented in Table 2. No statistically significant differences were observed between the lower and higher P4 groups in terms of BMI, endometrial thickness, peak LH and E2 levels on the day of LH surge, the number of single embryo transfers P4 levels on the day of ET, and β-hCG values. However, a significantly higher proportion of embryo transfers were performed on day 5 in patients with P4 levels above the threshold (*p* < 0.05) (Table 2). Both clinical pregnancy rates (73.8% vs. 14.9%) and ongoing pregnancy rates (63.0% vs. 12.8%) were significantly higher in the group with P4 levels above the threshold (*p* < 0.001).

**Table 2:** Comparison of the demographic characteristics and cycle features of the study population in HRT cycle FET based on the cutoff P level on the day of the pregnancy test.

	NC			HRT		
	P4 cutoff level on the β-hCG test day					
	< 15.05 n = 47	≥ 15.05 n = 80	*p* value	< 14.15 n = 232	≥ 14.15 n = 262	*p* value
Age (year)	34.3 ± 6.0	34.1 ± 5.6	0.868	33.2 ± 6.1	34.9 ± 6.2	** < 0.05**
BMI(kg/m^2^)	23.9 ± 4.4	24.4 ± 4.8	0.607	26.9 ± 5.3	24.6 ± 4.5	** < 0.001**
Duration of infertility (year)	4.6 ± 1.9	4.3 ± 2.1	0.394	4.6 ± 2.5	4.4 ± 2.2	0.244
Endometrial thickness (mm)	9.5 ± 1.6	10.2 ± 1 .9	0.054	9.3 ± 1.6	9.5 ± 1.5	0.198
Mean E_2_ (pg/mL)	294.2 ± 109.8	271.9 ± 108.7	0.274	269.3 ± 107.6	271.2 ± 121.5	0.854
Mean LH (mIU/ml)	42.9 ± 19.4	42.2 ± 22.6	0.866	16.3 ± 10.6	18.8 ± 12.5	** < 0.05**
*Embryo stage (%)*						
D3	12.8	7.5	** < 0.05**	19	22.9	**0.297**
D5	87.2	92.5		81	77.1	
Single ET (%)	68.1	50.0	0.136	65.1	59.9	0.237
Mean P4 on ET day (ng/mL)	16.2 ± 7.8	17.7 ± 8.7	0.334	18.6 ± 7.9	21.9 ± 10.1	** < 0.001**
Mean β-hCG (mIU/mL)	26.7 ± 56.2	218.9 ± 196.6	** < 0.001**	109.5 ± 163.0	150.1 ± 188.3	0.011
Mean P4 on β-hCG test day (ng/mL)	10.7 ± 2.8	29.1 ± 10.5	** < 0.001**	11.2 ± 2.1	25.4 ± 12.6	** < 0.001**
Clinical pregnancy rate (%)	14.9	73.8	** < 0.001**	34.9	53.1	** < 0.001**
Ongoing pregnancy rate (%)	12.8	63.0	** < 0.001**	31.9	48.1	** < 0.001**
Biochemical miscarriage rate (%)	11.6	8.3	0.561	10.8	6.4	0.127

Data are presented as mean ± standard deviation (SD), number (n), or percentage (%).

*HRT* Hormone Replacement Therapy, *NC* Natural Cycle, *P4* Progesterone, *β-hCG* Beta-human Chorionic Gonadotropin, *BMI* Body Mass Index, *E2* Estradiol, *LH* Luteinizing Hormone, *ET* Embryo Transfer, *D3 / D5* Embryo stage day (Day 3 / Day 5 embryo).

A *p*-value < 0.05 was considered statistically significant; significant values are shown in bold.

### Estimation of cut off P4 levels to predict ongoing pregnancy in HRT-FET cycles

**Fig. 2:**
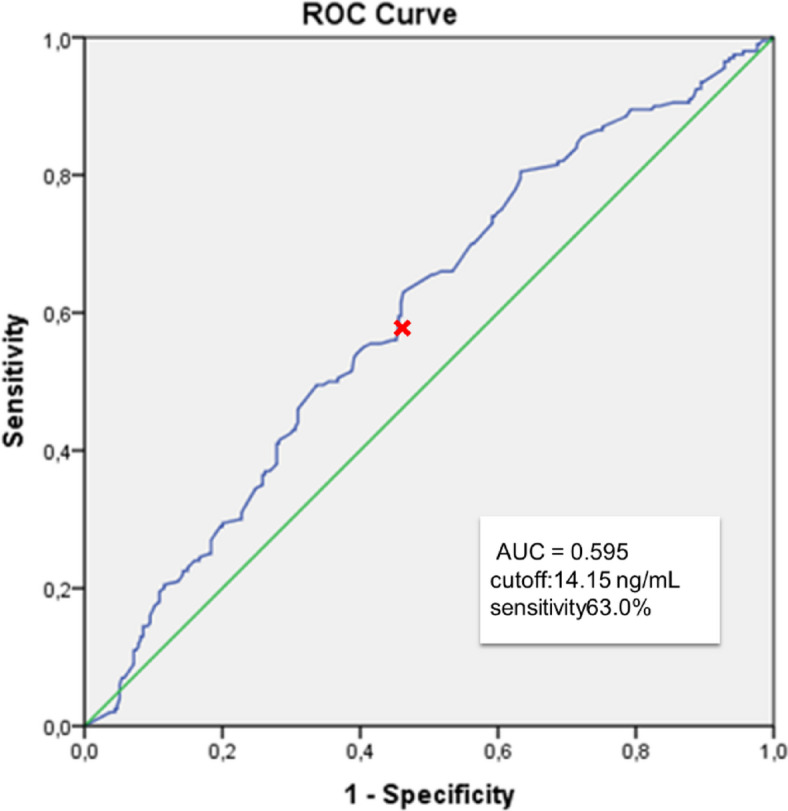
ROC analysis of serum progesterone levels on β-hCG day for predicting ongoing pregnancy in HRT-FET cycles.

In HRT cycles, ROC curve analysis revealed that the AUC was 0.595 (95% CI 0.545–0.646, *p* < 0.001) (Fig. 2) and the optimal serum P4 threshold on the day of the pregnancy test for predicting ongoing pregnancy was 14.15 ng/mL, with a sensitivity of 63.0% and a specificity of 53.7%.

In HRT cycles, 44.5% of patients had serum P4 levels below this threshold. When categorized accordingly, patients with P4 levels above the threshold were older (*p* < 0.05), had lower BMI (*p* < 0.001), higher serum LH levels before initiation of P4 support (*p* < 0.05), and higher P4 levels on the day of ET (*p* < 0.001) (Table 2). There were no statistically significant differences between patients above and below the threshold in terms of E2 levels on the day before P4 support, the percentage of single embryo transfers, or stage of the embryos. The mean β-hCG values in the HRT group were 109.5 ± 163.0 and 150.1 ± 188.3 in the lower and higher P4 groups, respectively (*p* < 0.05). Clinical pregnancy rates (53.1% vs. 34.9%) and ongoing pregnancy rates (48.1% vs. 31.9%) were significantly higher in the group with P4 levels above the threshold (*p* < 0.001) (Table 2).

Multivariable logistic regression analyses were performed separately for the HRT and NC groups to identify independent predictors of ongoing pregnancy (Table 3).

**Table 3:** Analysis of predictive factors for ongoing pregnancy rates using GEE regression for the HRT and NC groups.

Parameters	Natural Cycle			HRT Cycle		
	OR	95% CI	*p value*	OR	95% CI	*p value*
Age (years )	0.83	0.76–0.91	** < 0.05**	0.92	0.88–0.96	** < 0.001**
BMI				1.03	0.98–1.08	0.240
ET day = 3 (Ref: ET Day = 5)	0.45	0.10–2.12	0.311	0.27	0.13–0.59	** < 0.05**
ET Day 5	1 [Ref]			1 [Ref]		
LH on trigger day	–	–	–	0.99	0.97–1.01	0.359
Progesterone on ET day	–	–	–	0.98	0.95–1.01	0.145
Rescue progesterone supplementation on ET day (Ref: without rescue)	–	–	–	1.09	0.51–2.33	0.827
Serum progesterone below threshold on β-hCG test day	0.04	0.01–0.13	** < 0.001**	0.31	0.18–0.51	** < 0.001**
Serum progesterone above threshold on β-hCG test day (Ref)	1 [Ref]			1 [Ref]		
Vaginal progesterone use in natural cycle (Ref: None)	1.02	0.40–2.59	0.969	–	–	–

Odds ratios (ORs), 95% confidence intervals (CIs), and p-values are reported.

The following were used as reference categories: Day 5 embryo transfer (vs. Day 3), serum progesterone above threshold on the β-hCG test day (vs. below threshold), no rescue progesterone on ET day (vs. rescue supplementation), and no vaginal progesterone use in NC cycles (vs. use). Statistically significant values (*p* < 0.05) are shown in bold.

*OR* odds ratio, *CI* confidence interval, *BMI* body mass index, *HRT-FET* hormone replacement therapy frozen embryo transfer, *NC-FET* natural cycle frozen embryo transfer, *ET* embryo transfer, *E₂* estradiol, *P₄* progesterone.

In the HRT group, younger age (OR = 0.92, 95% CI 0.88–0.96, *p* < 0.001) was significantly associated with higher odds of ongoing pregnancy. In contrast, day 3 embryo transfer, compared to day 5, was associated with lower pregnancy rates (OR = 0.27, 95% CI 0.13–0.59, *p* < 0.05). Additionally, serum progesterone levels below the threshold on the β-hCG test day were significantly associated with reduced pregnancy rates (OR = 0.31, 95% CI 0.18–0.51, *p* < 0.001; reference group: above threshold).

In the NC group, younger age (OR = 0.83, 95% CI 0.76–0.91, *p* < 0.05) and serum progesterone levels above the threshold on the test day (OR for below threshold = 0.04, 95% CI 0.01–0.13, *p* < 0.001, reference group: above threshold) were significantly associated with higher ongoing pregnancy rates.

## Discussion

In this cohort study**,** we found that pregnancy outcomes improved above the serum progesterone thresholds measured on the day of pregnancy testing in patients undergoing FET with either HRT or natural cycle protocols**,** and a positive correlation was observed between serum P4 levels on the β-hCG day and ongoing pregnancy rates when analyzed by percentiles**.** Additionally, progesterone levels measured on the embryo transfer day were higher in patients with P4 levels above the threshold on the day of pregnancy testing**.** Our results support previous studies, although limited in number, that have reported similar findings.

While the significance of serum progesterone levels on the day of ET is well established, and rescue protocols have demonstrated improved pregnancy outcomes in HRT-FET cycles, the dynamics of progesterone levels after ET remain unclear. In a recent study, Özcan et al.^23^ demonstrated that 83% of patients with low serum P4 on the day of ET achieved adequate levels (≥ 10 ng/mL) after receiving rescue progesterone supplementation. Notably, 90% of pregnancies in patients who initially had serum progesterone levels < 10 ng/mL occurred in those who reached adequate concentrations following daily SCP treatment^23^.

In recent years, a few studies have investigated the impact of P4 levels measured on the day of β-hCG testing on treatment success of FET cycles. A prospective study conducted by Racca et al. in 2023 evaluated 664 FET cycles, focusing solely on cycles utilizing the HRT protocol. The threshold value for P4 levels on ET day was set at 10.6 ng/mL, and only patients with P4 levels above this threshold were included in the study. To achieve this threshold on the day of pregnancy testing, the required ET day P4 value was calculated using ROC curve analysis and determined to be 13.6 ng/mL. Pregnant patients were categorized into three groups based on their P4 levels on the day of β-hCG testing: those with P4 levels above the threshold, those with P4 ≤ 10.6 ng/mL who received an increased treatment dose, and those with P4 ≤ 10.6 ng/mL who continued with their initial treatment regimen. Live birth rates were reported as 71.9%, 96%, and 7.3% for these groups, respectively. A positive correlation was also found between ET day P4 and β-hCG day P4 levels^18^.This study demonstrated that low P4 values on the day of pregnancy test in HRT-FET cycles negatively impact pregnancy outcomes. A key difference between our study and Racca’s is that we included both natural and HRT protocols in separate and combined analyses. Unlike Racca’s study, the primary objective of our research was to evaluate the predictive value of P4 levels measured on the pregnancy test day for pregnancy outcome. Additionally, our study utilized both vaginal and subcutaneous progesterone, whereas Racca’s study exclusively focused on vaginal MVP. Patients whose P4 levels were below the threshold on ET day and had their doses increased were included in the current study. In Racca’s study, 30.4% of cases had P4 levels below 10.6 ng/mL on the β-hCG day, while this rate was 11.3% in our study. The difference may be attributed to the use of SCP in our study. However, both studies concluded that β-hCG day P4 levels were associated with pregnancy prognosis. In our study, a statistically significant weak positive correlation was observed between serum P4 and β-hCG levels on the day of testing (r = 0.184, *p* < 0.001), suggesting a modest association between luteal P4 levels and early trophoblastic activity.

A recent retrospective study by Alsbjerg et al. evaluated the optimal P4 value on the day of pregnancy testing and its impact on pregnancy outcomes in a relatively small number of FET cycles using the HRT protocol with estradiol valerate and micronized progesterone gel for endometrial preparation. A P4 threshold of 35 nmol/L (11 ng/mL) was established, with 51% of patients having P4 levels below this cut off. Adjusted for maternal age, BMI, smoking, number of embryos transferred and blastocyst age (day 5 or 6), the OR for ongoing pregnancy was significantly decreased (OR = 0.54, 95% CI 0.32–0.91, *p* = 0.02) when P4 was below the cut off^19^. These findings are compatible with our results. A notable difference is that in our study, mid-luteal P4 levels were measured before ET, and additional P4 supplementation was provided for values < 10 ng/mL. The cut off P4 for HRT was 14.15 ng/mL, which is higher than 11 ng/mL in Alsbjerg’s study. This difference can be explained by the fact that Alsbjerg’s study exclusively used vaginal MVP for luteal support, whereas our study included MVP and SC P4.

The main strength of our study was the comparison of patients undergoing both NC and HRT-FET protocols in relation to the impact of pregnancy test day P4 levels on pregnancy outcomes. Luteal phase deficiency (LPD), which affects around 8.1% of natural cycles^24^, has been implicated in infertility, subfertility, and early first trimester pregnancy loss. Overall, it remains unclear whether abnormal luteal function is an independent cause of implantation failure or early pregnancy loss in natural cycles^25^. Therefore, monitoring luteal phase hormone dynamics in natural and HRT cycles is recommended to optimize pregnancy outcomes. There is limited data available on luteal P4 levels in natural FET cycles. In our population of natural cycles, 3% of patients had serum P4 levels below 10 ng/mL, a cut off generally accepted as a cut off in HRT-FET cycles, on the day of ET and received additional P4 accordingly. In natural cycle FETs utilizing euploid embryos with luteal phase support, progesterone levels did not differ significantly between patients achieving ongoing pregnancy and those who did not, including when analyzed by stratified ranges (> 5–10, > 10–15, > 15–20, and > 20 ng/mL)^26^. In two recent meta-analyses, P4 supplementation was associated with higher pregnancy rates in true natural (tNC) FET but not in modified natural cycle (mNC) FET^27,28^. To our knowledge, this represents the first study to analyze P4 levels on ET day, implement a rescue P4 supplementation protocol, and evaluate post-intervention P4 levels measured on pregnancy test day with clinical outcomes. The cutoff P4 levels in natural cycles were higher than in HRT-FET in our study, likely due to the sufficient P4 secretion from the corpus luteum compared to exogenous luteal support. The higher sensitivity and specificity values observed in NC-FET cycles may also be attributed to the physiological function of the corpus luteum, which provides steady endogenous progesterone secretion and a more synchronized endometrial environment for successful implantation. This finding supports the choice of natural cycles in FET. No supplemental progesterone was administered in cases with low P4 levels on the day of pregnancy testing, the observed reduction in ongoing pregnancy rates below the threshold in the current study strongly suggests that implementing a rescue protocol after pregnancy confirmation may improve outcomes in both HRT and natural cycle FET pregnancies.

A major limitation of this study is the absence of a control group that did not receive progesterone rescue for low P4 levels on the day of embryo transfer, given that the clinical benefit of such supplementation has been demonstrated in previous studies. In line with our study, Racca et al. included patients with P4 levels ≥ 10.6 ng/mL on the day before FET. In contrast, Alsbjerg’s study administered a standard P4 dose to all patients without any adjustments based on P4 levels on the day of embryo transfer^18,19^. For that reason, the cut off level of 35 nmol/L (11 ng/mL) on the day of the pregnancy test corresponds to P4 levels in a cohort of HRT-FET patients who received a standard P4 dose without individualized luteal support adjustments based on P4 levels on the day of FET. In fact, since the current accepted treatment approach involves individualizing the P4 dose based on P4 levels on the day of embryo transfer, our approach provides meaningful insight compared to standard luteal support without P4 monitoring. Additionally, the potential confounding effects of patient heterogeneity were addressed by incorporating age, BMI, duration of infertility and embryo stage into the regression model. The developmental stage of the embryo may have also impacted the outcomes; as indicated in the regression analysis, blastocyst transfers were associated with higher ongoing pregnancy rates, likely reflecting better embryo selection and optimal synchronization between the endometrium and the embryo.

In conclusion, the strategy to measure P4 levels on the β-hCG testing day in natural and HRT-FET cycles provides valuable information regarding pregnancy outcomes, and individualizing by a rescue P4 dose in patients with low P4. Our findings further highlight the clinical significance of monitoring P4 levels on the day of ET in natural cycles, supporting the potential benefit of exogenous P4 supplementation as a rescue strategy when levels are suboptimal. However, further studies are needed to determine the optimal P4 cutoff level on the day of embryo transfer in tNC protocols. This study demonstrates that suboptimal P4 levels during early pregnancy are associated with adverse pregnancy outcomes in FET cycles, with consistent effects observed in both natural and HRT cycles. Given the availability of diverse P4 supplementation methods in HRT-FET cycles, establishing P4 threshold values across a comprehensive range of administration routes would enhance clinical applicability.

## Data Availability

The datasets generated and/or analyzed during the current study are available from the corresponding author on reasonable request. Deidentified individual participant data will also be made available to editors upon request.
